# Regionally-derived cell populations and skeletal stem cells from human foetal femora exhibit specific osteochondral and multi-lineage differentiation capacity *in vitro* and *ex vivo*

**DOI:** 10.1186/s13287-015-0247-2

**Published:** 2015-12-18

**Authors:** David Gothard, Kelvin Cheung, Janos M. Kanczler, David I. Wilson, Richard O. C. Oreffo

**Affiliations:** Bone and Joint Research Group, Centre for Human Development, Stem Cells and Regeneration, Human Development and Health, University of Southampton, School of Medicine, Institute of DevelopmentalSciences, Mail Point 887, Southampton General Hospital, Tremona Road, Southampton, SO16 6YD UK; Centre for Human Development, Stem Cells and Regeneration, Human Development and Health, University of Southampton, School of Medicine, Institute of Developmental Sciences, Mail Point 887, Southampton General Hospital, Tremona Road, Southampton, SO16 6YD UK; University Hospital Southampton NHS Foundation Trust, Tremona Road, SO16 6YD Southampton, UK

**Keywords:** Adipogenesis, Bone, Chondrogenesis, Development, Diaphysis, Epiphysis, Human foetal femur, Osteogenesis, Stro-1

## Abstract

**Background:**

Adult skeletal stem cells (SSCs) often exhibit limited *in vitro* expansion with undesirable phenotypic changes and loss of differentiation capacity. Foetal tissues offer an alternative cell source, providing SSCs which exhibit desirable differentiation capacity over prolonged periods, ideal for extensive *in vitro* and *ex vivo* investigation of fundamental bone biology and skeletal development.

**Methods:**

We have examined the derivation of distinct cell populations from human foetal femora. Regionally isolated populations including epiphyseal and diaphyseal cells were carefully dissected. Expression of the SSC marker Stro-1 was also found in human foetal femora over a range of developmental stages and subsequently utilised for immuno-selection.

**Results:**

Regional populations exhibited chondrogenic (epiphyseal) and osteogenic (diaphyseal) phenotypes following *in vitro* and *ex vivo* characterisation and molecular analysis, indicative of native SSC maturation during skeletal development. However, each population exhibited potential for induced multi-lineage differentiation towards bone (bone nodule formation), cartilage (proteoglycan and mucopolysaccharide deposition) and fat (lipid deposition), suggesting the presence of a shared stem cell sub-population. This shared sub-population may be comprised of Stro-1+ cells, which were later identified and immuno-selected from whole foetal femora exhibiting multi-lineage differentiation capacity *in vitro* and *ex vivo*.

**Conclusions:**

Distinct populations were isolated from human foetal femora expressing osteochondral differentiation capacity. Stro-1 immuno-selected SSCs were isolated from whole femora expressing desirable multi-lineage differentiation capacity over prolonged *in vitro* expansion, superior to their adult-derived counterparts, providing a valuable cell source with which to study bone biology and skeletal development.

**Electronic supplementary material:**

The online version of this article (doi:10.1186/s13287-015-0247-2) contains supplementary material, which is available to authorized users.

## Background

Bone damage and loss due to disease and trauma is a growing concern for an increasingly aged population. Many methods exist to repair bone defects including auto- and allografts, and *de novo* tissue engineering [1–4]. However, a major limitation of reparative strategies is fully understanding the biological mechanisms controlling skeletal development. Elucidation of native development and healing would inevitably improve successful bone defect treatment, whether by reparation, replacement or regeneration. To investigate basic human bone biology and understand the fundamental mechanisms of bone formation and repair, a robust *in vitro*/*ex vivo* developmental paradigm representative of native skeletal development is required. A critical step in this approach is the identification and isolation of osteoprogenitor cells capable of osteochondral differentiation to inform bone regeneration and augmentation [5–7]. Furthermore, a homogeneous bone stem cell population would enable interrogation of skeletal development and aid screening for reparative strategies, including osteoconductive scaffolds and osteoinductive signalling molecules and mechanical cues [8, 9].

Adult human bone marrow stromal cells (HBMSCs) contain a diminutive bone stem cell population (1 in 10,000 to 50,000) often referred to as mesenchymal stem cells (MSCs), which exhibit osteochondral differentiation capacity [10–12]. MSCs have been shown in a number of studies to generate tissues of the *in vivo* musculoskeletal system including bone, cartilage, fat, ligament, muscle and tendon [10, 13–17]. However, conventional MSC populations are highly heterogeneous and controversy over their exact identity and differentiation potential remains with reports of hepatocyte and neuronal differentiation [18, 19]. The observed heterogeneity [20, 21] is likely a consequence of their derivation from a multitude of adult tissues including dental pulp, fat, muscle, skin, synovium [22–27], and extra-embryonic tissues including amniotic fluid, placenta and umbilical cord blood [28–31]. This highlights the need for robust *in vitro* clonal analysis and *in vivo* validation of exact differentiation capacity. The exact phenotype is more than scientific pedantry as heterogeneity impacts on MSC suitability for clinical application, demanding an additional purification step either before controlled differentiation (isolation of a homogeneous stem cell population) [6] or following heterogeneous differentiation (isolation of specific terminally differentiated cell types) [32]. Given the imprecise nature and often misappropriate use of the term MSC (typically almost any adherent fibroblastic cell population, as well as the observation that MSCs from different tissues are not the same), we have used the term skeletal stem cell (SSC) [2, 33–35] in reference to the stem cell population with specific differentiation capacity (haematopoietic supportive stroma) towards all skeletal tissues including bone, cartilage, and fat [9, 36–38].

An alternative cell source to adult tissue, yet controversial, is foetal tissue which has been shown to contain cell populations with comparable if not enhanced reparative function [39–42]. Foetal femora are composed of proliferative osteochondral progenitor cells capable of self-renewal, differentiation and bone and cartilage formation. During development, these populations exhibit regional differences driving endochondral ossification and formation of long bones. The epiphyseal region is predominantly cartilaginous, whilst the diaphyseal region undergoes mineralisation and bone formation. Cell populations isolated from these separate regions would help delineate the sequential biological mechanisms driving bone formation and inform efforts to improve bone repair and regeneration. As in adult tissues, foetal SSCs constitute osteochondral progenitors capable of cartilage and bone formation, and the authors hypothesise that both epiphyseal and diaphyseal populations share this SSC sub-population.

One surface marker which has shown robust enrichment specificity for SSCs is trypsin-resistant cell surface antigen 1 (Stro-1) [43–46]. Adult Stro-1+ populations exhibit enhanced colony forming unit–fibroblastic (CFU-F) capacity and elevated osteogenic differentiation both *in vitro* and *in vivo* in comparison to unsorted adult HBMSCs [47–50]. Consequently, Stro-1 expression was sought in human foetal femora to identify possible foetal SSCs. Previous work has shown that whole foetal femur-derived cell populations offer an alternative to adult populations, specifically for *in vitro* investigation of human skeletal development, displaying significantly enhanced proliferation and multi-lineage differentiation capacity [5, 51–55]. Furthermore, studies have demonstrated significant enhancement of bone defect repair following application of whole foetal femur-derived cell populations in biodegradable carrier scaffolds [56–59].

The current study has isolated and characterised a Stro-1+ sub-population in whole foetal femora, representing foetal femur-derived SSCs. Specifically, we have examined the potential of foetal femur tissue as a source of inducible SSCs, built on the premise that distinct regional cell populations could be routinely derived exhibiting specific chondrogenic and osteogenic differentiation capacity, and that Stro-1 enrichment could target a SSC sub-population. These populations could provide the necessary *in vitro/ex vivo* investigative tools to elucidate bone biology mechanisms active during skeletal development.

## Methods

All tissue culture reagents, growth factors, chemicals and other materials were supplied by Sigma Aldrich unless otherwise stated. Collagen type I polyclonal antibody was a kind gift from Dr. Larry Fisher (National Institutes of Health (NIH)). Hydroxyapatite-loaded poly-lactic acid scaffolds (HA-PLA) were kindly provided by Dr. Lisa White, University of Nottingham. Stro-1 hybridoma was re-derived from an original donation provided by Dr. Jon N. Beresford, University of Bath [44, 60]. Fertilized chicken eggs were supplied by Henry Stewart & Co. Ltd, Fakenham, Norfolk, NR21 8LZ, United Kingdom.

### Ethics statement

Foetal tissue was obtained following informed consent and ethical approval from the National Research Ethics Committee South Central-Southampton (REC 296/00296100). All animal procedures were carried out in accordance with the guidelines and regulations laid down in the Animals (Scientific Procedures) Act 1986. As part of the application for a project licence under which to perform the animal work, animal procedures in this study were reviewed and approved by the Scientific Review Group, part of the Animal Welfare and Ethical Review Body at the University of Southampton. Chick embryos were sacrificed at embryonic day 11 by schedule 1 decapitation according to Home Office Approval UK (Project license – PPL 30/2762). Femora were dissected from embryonic day 11 chick embryos (*Gallus domesticus*), and soft tissue was carefully removed.

### Cell isolation and culture

#### Human foetal femur preparation

Femora were obtained following termination of pregnancy and informed patient consent. Foetal age was determined according to foot length and expressed as days post-conception. Samples ranged from 53 to 69 days (4 mm to 8.5 mm) [5, 61]. Femora were dissected and surrounding soft tissues were removed by gentle rolling back and forth across sterile filter paper. Soft tissues stuck to the filter paper whilst the femur was separated cleanly. Dependent on the developmental stage of each sample, the periosteum was removed manually by dissection (older samples) or as part of the preparation procedure (younger samples).

#### Epiphyseal and diaphyseal cell isolation

Prepared femora were dissected into epiphyseal and diaphyseal regions via a transverse incision through the metaphysis region at either end of the bone collar (Additional file 1: Figure S1). The bone collar distinguished the boundary between the diaphysis and epiphyses. Both proximal and distal epiphyses were combined in each sample and carefully cut into small segments, as was the diaphysis separately, prior to overnight collagenase B (Roche Products Limited (Pharmaceuticals), Welwyn Garden City, AL7 1TW, United Kingdom) digestion (1 mg/mL in minimum essential medium – alpha [α-MEM, Life Technologies Ltd, Thermo Fisher Scientific, Paisley, PA4 9RF, United Kingdom]) at 37 °C. Isolated cells were cultured in basal medium.

#### SSC isolation

Whole femora were cut into small segments and collagenase B digested overnight in preparation for Stro-1 immuno-selection. Most of the tissue was completely digested, however, individual isolated cells were passed through a 40 μm sieve (BD Biosciences, Oxford Science Park, Oxford OX4 4DQ, United Kingdom) to remove large clumps and mineralised bone collar, and suspended in basal medium (α-MEM, 10 % foetal calf serum (FCS, Invitrogen, Thermo Fisher Scientific, Paisley, PA4 9RF, United Kingdom), penicillin (100 U/mL, PAA Laboratories Ltd, Yeovil, Somerset, BA22 8YG, United Kingdom), and streptomycin (0.1 mg/mL, PAA Laboratories Ltd, Yeovil, Somerset, BA22 8YG, United Kingdom)). Cells were initially incubated with blocking buffer (α-MEM, 10 % human serum, 5 % FCS and 1 % bovine serum albumin (BSA, PAA Laboratories Ltd, Yeovil, Somerset, BA22 8YG, United Kingdom)) before incubation with primary Stro-1 antibody (undiluted hybridoma culture supernatant) [62]. Suspensions were subsequently washed three times in isolation buffer (2 mM ethylenediaminetetraacetic acid [EDTA] and 1 % BSA in phosphate buffered saline [PBS]) before incubation with magnetic bead-conjugated secondary antibody (200 μL in 1 mL isolation buffer, Miltenyi Biotec Ltd, Woking, Surrey GU24 9DR, United Kingdom). Samples were then washed and target cells isolated by magnetic-activated cell sorting (MACS) prior to suspension and culture in basal medium.

#### Adult SSC isolation

Adult Stro-1 populations were isolated as detailed above. In brief, bone marrow was isolated from haematologically normal osteoarthritic patients undergoing total hip replacement at the Southampton General Hospital with informed consent (approval from the Southampton and South West Hampshire Local Research Ethics Committee [LREC194/99]). Bone marrow was sieved to remove blood clots and bone fragments and treated with a density gradient solution (Lymphoprep™, Lonza, Slough, Berkshire, SL1 4DX, United Kingdom) to remove erythrocytes. The remaining cell suspension was then immuno-labelled with Stro-1 and sorted by MACS. Control unsorted cell populations were separated from each sample before Stro-1 antibody labelling. Unsorted cells were processed in parallel in PBS. Isolated cell populations were suspended and cultured in basal medium.

### Cell characterisation

#### Alkaline phosphatase expression

Cultures (P1) were PBS washed 3 h after seeding into culture flasks to remove non-adhered cells, and incubated for 14 days without basal medium change at 37 °C, 5 % CO_2_ in a humidified atmosphere. Cultures were fixed with 85 % ethanol before air drying then incubation with Fast Violet B salt (2.5 μg/mL) and Naphthol AS-MX phosphate (40 μL/mL) in dH_2_O for 30-45 min at 37 °C and 5 % CO_2_ in a humidified atmosphere under dark conditions. Cultures were then washed with H_2_O and counterstained in haematoxylin for 5 min. Cultures were washed with H_2_O again and left to air dry prior to quantification.

Seeding densities were selected based on whether clear colonies could be observed following qualitative assessment of a series of seeding densities including 0.5 × 10^1^, 1 × 10^1^, 1 × 10^2^, and 1 × 10^3^ cells/cm^2^.

#### Specific alkaline phosphatase activity

Samples were fixed in 85 % ethanol before treatment with 0.05 % Triton™ X-100 and three freeze-thaw cycles. DNA quantification was assessed by diluting cell lysate with Tris/EDTA buffer and addition of Quant-iT™ PicoGreen® dsDNA reagent (Life Technologies). Alkaline phosphatase (ALP) activity was assessed by diluting cell lysate in ALP substrate solution (2 mg/mL p-nitrophenyl phosphate [pNPP] in 0.75 M alkaline buffer solution). Samples were then incubated at 37 °C in the dark, under gentle agitation, and the reaction was terminated with 1 M sodium hydroxide after 45-60 min. Spectrophotometry was used to quantify DNA and ALP activity at 530 nm and 405 nm, respectively. ALP concentration was measured in nmol pNPP/mL h-1, and ALP activity in nmol pNPP/μg DNA.

### Differentiation culture – monolayer/micromass

#### Osteogenesis

Cells (P2) were seeded at 1 × 10^3^ cell/cm^2^ and cultured in osteogenic medium (α-MEM, 10 % FCS, 10 nM dexamethasone [Dex] and 100 μM ascorbate-2-phosphate [Asc]) for 14 days. Osteogenic medium was supplemented with 10 mM β-glycerophosphate to enable bone nodule formation. Cultures were fixed in 4 % paraformaldehyde (PFA) and stained with Alizarin red (1 % w/v in dH_2_O).

#### Chondrogenesis

A total of 2.5 × 10^5^ cells (P2) per micromass were suspended in 10-20 μL basal medium, pipetted carefully into a six-well plate and incubated for 1 h allowing attachment to the plate and cell/cell adhesion. Basal medium was removed and chondrogenic medium (α-MEM, 10 nM Dex, 100 μM Asc, 10 ng/mL TGFβ-3 [Peprotech, London, W6 8LL, United Kingdom]) and 10 μg/mL insulin (in the form of 100× insulin/tranferrin/sodium selenite [ITS] solution) was carefully added so as to not disturb the micromass. After 21 days, cultures were fixed in 4 % PFA and stained with Alcian blue (0.5 % w/v in acidic H_2_O [0.01 % acetic acid]).

#### Adipogenesis

Cells (P2) were seeded at 1 × 10^3^ cell/cm^2^ and cultured in adipogenic medium (α-MEM, 10 % FCS, 1 μM Dex, 0.5 mM 3-isobutyl-1-methylxanthine [IBMX], 100 μM indomethacin and 10 μg/mL ITS solution) for three days followed by one day in ITS only medium (α-MEM, 10 % FCS and 10 μg/mL ITS solution). This was repeated three times before continuous culture in ITS medium up to 28 days. Cultures were fixed in Baker’s formal calcium (0.1 g/mL calcium chloride in 4 % formaldehyde), rinsed with 60 % isopropanol, and stained with a filtered working solution (3:2 in dH_2_O) of stock Oil Red O (powder saturated 99 % isopropanol) for 15 min. Alternative adipogenic medium (α-MEM, 10 % FCS, 100 nM Dex, 0.5 mM IBMX, 3 μg/mL ITS solution and 1 μM rosiglitazone) was utilised for later differentiation cultures. Revised adipogenic medium was observed to induce lipid formation within 14 days.

All cultures received medium changes twice weekly. All differentiation media were not supplemented with penicillin and streptomycin. Following revised adipogenic medium, all diaphyseal and epiphyseal differentiation cultures were revised to a total 14 day culture period.

### 3D pellet formation and organotypic culture

Epiphyseal and diaphyseal populations were cultured as pellets (2.5 × 10^5^ cells per pellet [P2]) in basal medium for 48 h to allow formation before transfer to organotypic culture on polytetrafluoroethylene (PTFE) confetti membranes (BioCell Interface, La Chaux-de-Fonds, Switzerland) on tissue culture flask-well inserts. Organotypic pellets were cultured for 21 days in either basal, osteogenic or chondrogenic medium (detailed previously). After 21 days, pellets were processed and assessed for ALP activity as described previously.

### Histomorphometry analysis

Following differentiation culture and histological staining, three or five representative images (unsorted vs Stro-1, and epiphyseal vs diaphyseal, respectively) across each sample well (triplicate wells) were used to quantify differentiation. CellProfiler image software was employed to quantify the area (cm^2^) of stain per 10^5^ cells (cell nuclei were counterstained with DAPI and imaged by fluorescence microscopy). Following micromass culture in chondrogenic medium, histological staining and imaging, Fiji (Image J) image software was used to quantify mean colour intensity in individual micromass pellets (triplicate wells per sample: one to three micromass pellets per well, epiphyseal vs diaphyseal, and unsorted vs Stro-1, respectively). Colour quantification was achieved through greyscale conversion and assessment of mean intensity across the diameter of the micromass pellet (0 to 255 scale; black through to white): values were inverted so 0 was white and 255 was black.

### Molecular analysis

MirVana™ RNA Isolation System Kit (Life Technologies) was used for RNA extraction according to the manufacturer’s protocol following 14 days culture in basal conditions. Cultured samples were placed on ice and washed twice in PBS. Lysis buffer and a homogenizing agent were used to liberate RNA from cells, followed by acid phenol-chloroform. The resultant mixture was centrifuged to allow phase separation. The RNA-containing aqueous phase was removed and added to ethanol prior to elution through a spin column. The column was washed three times with supplied buffer solutions and RNA eluted in RNase free water.

For cDNA synthesis, SuperScript® VILO cDNA Synthesis Kit (Life Technologies) was used for cDNA synthesis. RNA was combined with 5x VILO™ reaction mix and 1 μL of 10x SuperScript® enzyme was added to RNA samples and incubated at 25 °C for 10 min followed by 42 °C for 2 hr. The reaction was terminated by incubation at 85 °C for 5 min. Then, 40 μL of ultra-pure H_2_O (upH_2_O) was added to the cDNA sample to give a 1:4 dilution and stored at -20 °C or used immediately for quantitative RT-qPCR analysis. Quantitative RT-qPCR was performed using SYBR-Green PCR master mix (Life Technologies): 10 μL of SYBR-Green master mix; 5 μL of upH_2_O; 2 μL of forward and reverse primers for the gene of interest (Additional file 2: Table S1) and 1 μL of cDNA sample. The final mixture (20 μL) was added to each well of a 96-well-plate, analyzed using an Applied Biosystem (Life Technologies), 7500 Real Time PCR system. Data generated by RT-qPCR were analysed using the Applied Biosystem 7500 System SDS Software, version 2.0.5. Ct value (cycle threshold) for each sample was normalized to β-actin, an endogenous housekeeping gene, and fold expression levels for each target gene were calculated using the delta-delta Ct method.

### *Ex vivo* tissue regeneration

#### Chick femora isolation

Fertilized eggs were incubated (Hatchmaster, Brinsea Products Ltd, Weston Industrial Estate, Weston Super Mare, BS24 9BG, United Kingdom) for 11 days. Chick embryos were euthanized by decapitation prior to bilateral dissection of femora. Surrounding soft tissue was removed from the femora before overnight incubation in basal medium [63, 64].

#### Defect preparation and 3D pellet implantation

Following test defects using a 300 μm diameter drill (Additional file 3: Figure S2), a diaphyseal defect was positioned centrally along the length of the femur, and epiphyseal defects were positioned halfway between the end of the femur and the end of the bone collar. The femurs continue to grow and the tissues swell during the 10-day culture period contributing to partial closure of the defects. However, the defects are still visible after 10 days without implantation of cell pellets. Drill defects were plugged with appropriate preformed cell pellets (1.5 × 10^4^ cells [P2] per pellet, centrifuged at 1,000 rpm for 4 min, and incubated for 48 h in basal medium) and cultured organotypically on well inserts for 10 days in α-MEM with 100 μM Asc. Medium was changed every day before 4 % PFA fixation. A standard curve detailing the correlation between cell number (1 × 10^4^, 5 × 10^4^, 1 × 10^5^, 2.5 × 10^5^, 5 × 10^5^, and 1 × 10^6^ cells) and pellet diameter was used to assess the appropriate cell number required to generate a 300 μm diameter pellet (Additional file 4: Figure S3).

*Ex vivo* chick femur defects offered a simple and relatively rapid model for assessment of differentiation and tissue regeneration capacity inherent to distinct isolated populations. The advantage of the system is that it offers a high throughput skeletal model for screening candidate cell populations prior to expensive and time consuming *in vivo* models. Previous work in the group has shown that organotypic culture beyond 10 days has detrimental effects on cell viability in the femora. Embryonic day 11 chick femora were selected due to the presence of an osteogenic phenotype dominating the diaphyseal region with bone collar formation, and a chondrogenic phenotype dominating the epiphyseal regions. Both environments were required to assess the osteochondral differentiation capacity of implanted pellets.

### Histological analysis

#### Sample preparation and sectioning

Pellets and organotypic femora were fixed in 4 % PFA for 24 h, dehydrated through a series of ethanol washes (50 %, 70 %, 90 % in dH_2_O, and 2 × 100 %, 1 h in each), and incubated in Histo-Clear (National Diagnostics UK, Unit 4 Fleet Business Park, East Riding of Yorkshire, HU139LX, United Kingdom) for 1 h prior to paraffin wax at 60 °C (1 h). Processed samples were subsequently embedded in wax blocks using an automated Shandon Citadel 2000 ready for histological assessment. Embedded samples were sectioned at a thickness of 7 μm using a Microm HM330 D-6900 microtome (Heidelberg Instruments, Tullastraße 2, 69126 Heidelberg, Germany). All sections from pellets and femora were collected. Sections were stained, washed in water, dehydrated where appropriate and mounted with DPX before imaging on an Olympus BX-51/22 dotSlide digital virtual microscope using OlyVIA 2.1 software (Olympus Soft Imaging Solutions GmbH, Johann-Krane-Weg, 3948149 Münster, Germany, GmBH).

#### Alcian blue/Sirius red

Slides were de-waxed and rehydrated before treatment with haematoxylin (10 min), followed by acid-alcohol dip and treatment with Alcian blue (10 min – 0.5 % w/v), molybdophosphoric acid (10-20 min – 1 % w/v) and Sirius red (45-60 min - 0.1 % w/v in 100 mL picric acid and 200 mL dH_2_0).

#### Von Kossa

Slides were de-waxed and rehydrated before treatment with 1 % silver nitrate under UV irradiation for 20 min. Slides were then treated with 2.5 % sodium thiosulfate (8 min) and counterstained with Alcian blue (1 min) and van Gieson (5 min – 0.9 mg/mL Acid Fucshin in 50 % Picric acid).

#### Goldner’s Trichrome

Slides were de-waxed and rehydrated before treatment with haematoxylin (10 min), followed by acid/alcohol dip. Slides were treated for 5 min with 10 % v/v ponceau-fuchsin (0.75 % w/v and 0.25 % w/v, respectively, in 1 % acetic acid) and 2 % v/v azophloxin solution (0.5 % w/v in 0.6 % acetic acid) in acetic acid (0.2 % in dH_2_O). Slides were subsequently treated with 0.6 % w/v phosphomolybdic acid and 0.4 % w/v orange G solution in dH_2_O with thymol (20 min) before counterstaining with 0.2 % w/v light green solution in 0.2 % acetic acid. Stained sections were blotted dry and directly mounted with DPX.

#### Birefringence

Organotypic femora following Alcian blue/Sirius red (A/S) staining were also imaged using polarising filters to assess birefringence in implanted pellets by light microscopy. Femora were preferentially sectioned in the longitudinal plane to enable defect visualisation in the same section. Sections were oriented to follow the path of the drill defect through the femur to aid implant localisation. Birefringence colour correlated with collagen fibre alignment. Highly aligned collagen fibres in mineralised bone appeared red, whilst less aligned collagen fibres in pre-mineralised bone appeared yellow, and in osteoid appeared green.

### Immunochemistry

Sample sections were de-waxed and rehydrated before hydrogen peroxide (3 % in dH_2_O) quenching of endogenous peroxidase activity. Sections were incubated with blocking buffer (1 % BSA in PBS) for 15 min then primary antibody solution (1:1,000 COL1A1 (LF69), 1:500 COL2A1 (Cabiochem, Merck Millipore Ltd, Feltham, Middlesex, TW14 8NX, United Kingdom) and 1:50 SOX9 [Abcam, Cambridge Science Park, Cambridge, CB4 0FL, United Kingdom]) overnight at 4 °C. Biotinylated secondary antibody (1:200 in blocking buffer) was applied for 1 h before incubation with avidin-conjugated peroxidase. Finally, sections were treated with 3-amino-9-ethylcarbazole for 10 min until visualisation of a red-brown reaction product [5]. Sections were washed between steps with blocking buffer and mounted with Fluoromount™ once stained. Negative controls were run alongside lacking the respective primary antibody before imaging on dotSlide.

### Statistical analysis

A non-parametric Mann Whitney or Wilcoxon test was employed to assess significance between unpaired and paired data sets, respectively. Results are presented as mean ± SD. Significance is depicted by ^*^ P ≤ 0.05, ^**^ P ≤ 0.01, ^***^ P ≤ 0.001.

## Results

### Characterisation of foetal femur cell populations isolated by region

#### 2D characterisation of epiphyseal and diaphyseal populations *in vitro*

**Fig. 1:**
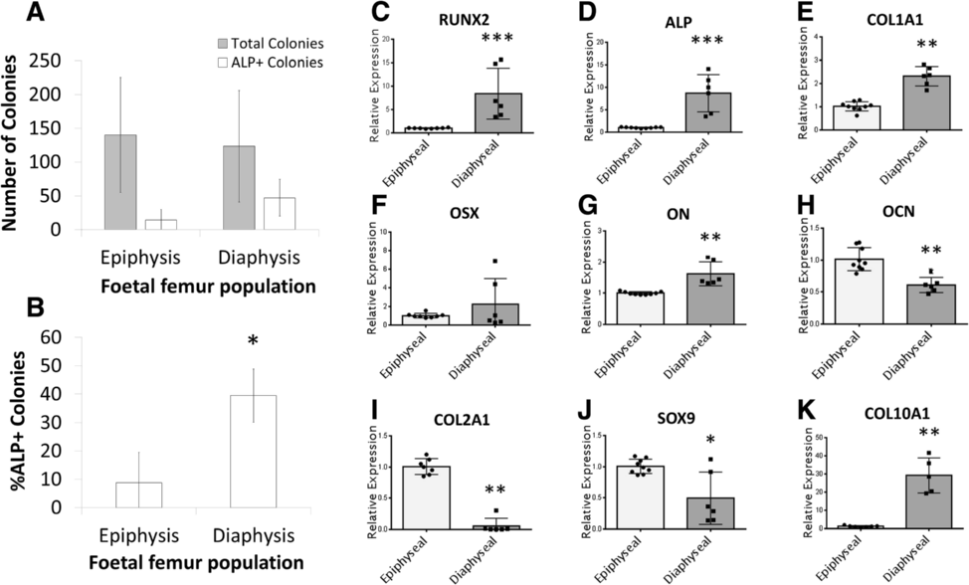
ALP expression and molecular analysis of epiphyseal and diaphyseal populations. Isolated cell populations (P1) were seeded into CFU-F assay (1 × 10^1^ cells/cm^2^) and cultured for 14 days prior to quantification of ALP+ colonies **a** and **b**. Populations were cultured in T25 cm^2^ flasks, and colonies were counted from the whole flask. ALP+ colonies are presented as a percentage of the total number of colonies. Molecular analysis in epiphyseal and diaphyseal populations assessed expression of osteochondral genes after 14 days culture in basal medium, including OSX **c**, RUNX2 **d**, ALP **e**, COL1A1 **f**, ON **g**, OCN **h**, COL10A1 **i**, SOX9 **j**, and COL2A1 **k**. Error bars are SD. * P ≤ 0.05, ** P ≤ 0.01, *** P ≤ 0.001 (foetal samples, n = 3; 55 61 and 63 days post conception). *ALP* alkaline phosphatase, *CFU-F* colony forming unit – fibroblastic

Following isolation and *in vitro* culture, both epiphyseal and diaphyseal populations exhibited similar colony formation capacity (Fig. 1a). However, diaphyseal populations exhibited significantly (P ≤ 0.05) enhanced ALP+ colony formation with an approximate three-fold increase (Fig. 1b).

Basal cultures of epiphyseal and diaphyseal populations were assessed by molecular analysis for a selection of osteochondral genes (Fig. 1). Diaphyseal populations exhibited significantly increased expression of osteogenic genes including RUNX2 (8.4 fold, P ≤ 0.001), ALP (8.6 fold, P ≤ 0.001), collagen type 1 (COL1A1, 2.3 fold, P ≤ 0.01) and osteonectin (ON, 1.6 fold, P ≤ 0.01) (Fig. 1c to f, respectively). Osterix (OSX) expression appeared greater in diaphyseal populations, although this did not reach significance (Fig. 1g). Osteocalcin (OCN) expression, a late osteogenesis marker, was significantly (P ≤ 0.01) reduced in diaphyseal compared to epiphyseal populations (Fig. 1h). Early chondrogenesis marker expression was significantly reduced in diaphyseal populations for genes including SOX9 (1.5 fold, P ≤ 0.05) and collagen type 2 (COL2A1, 20 fold, P ≤ 0.01) (Fig. 1i and j). Interestingly, collagen type 10 (COL10A1) expression, a late chondrogenesis marker, was significantly (P ≤ 0.01) increased in diaphyseal populations (Fig. 1k).

**Fig. 2:**
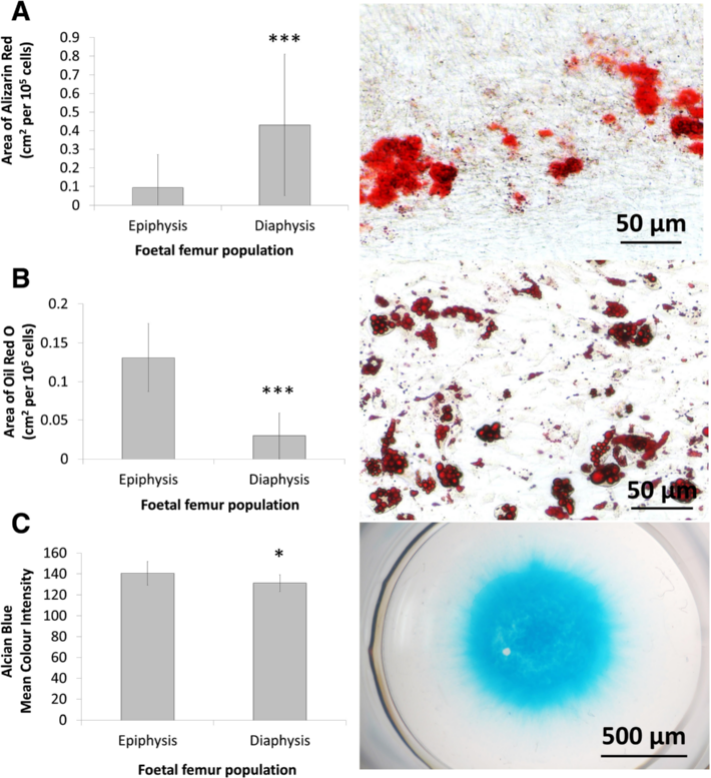
Multi-lineage differentiation potential of epiphyseal and diaphyseal populations. Monolayer cultures (P2) were seeded at 1 × 10^3^ cell/cm^2^, treated with osteogenic and adipogenic (rosiglitazone) medium for 14 days and stained with Alizarin red **a** and Oil Red O **b**, respectively. Micromass cultures (2.5 × 10^5^ cells per pellet) were treated with chondrogenic medium for 14 days and stained with Alcian blue **c**. Error bars are SD. * P ≤ 0.05, ** P ≤ 0.01, *** P ≤ 0.001 (foetal samples, n = 3; 53, 54 and 63 days post conception)

High ALP expression in diaphyseal populations correlated with significantly (P ≤ 0.001) increased calcification (Alizarin red stained bone nodule formation) and significantly (P ≤ 0.001) decreased lipid deposition (Oil Red O stained lipid deposition) (Fig. 2a and b, respectively) following differentiation culture. Cartilaginous matrix formation was also significantly (P ≤ 0.05) decreased in diaphyseal compared to epiphyseal populations (Alcian blue stained proteoglycan/mucopolysaccharide deposition) (Fig. 2c).

#### 3D characterisation of epiphyseal and diaphyseal populations *in vitro*

**Fig. 3:**
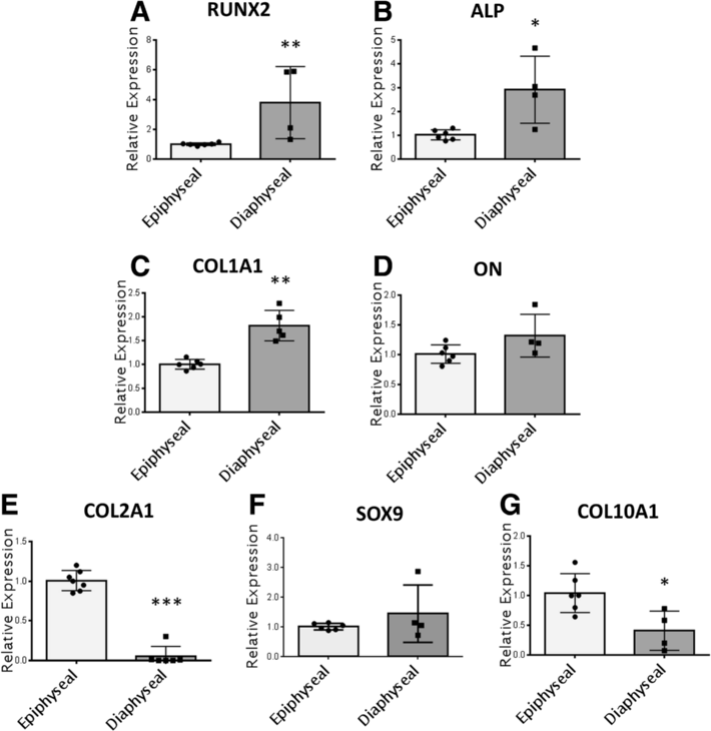
Molecular analysis of gene expression in epiphyseal and diaphyseal populations cultured as 3D pellets. Pellets (2.5 × 10^5^ cells per pellet) were cultured in basal medium for 21 days prior to analysis of osteochondral genes including RUNX2 **a**, ALP **b**, COL1A1 **c**, ON **d**, COL10A1 **e**, SOX9 **f** and COL2A1 **g**. Error bars are SD. * P ≤ 0.05, ** P ≤ 0.01 (foetal samples, n = 3; 55, 56 and 63 days post conception)

Diaphyseal 3D pellets exhibited significant (P ≤ 0.05) upregulation of osteogenic genes including ALP (2.9 fold), COL1A1 (1.8) and RUNX2 (3.8 fold) compared with epiphyseal 3D pellets (Fig. 3a to c). Results were similar to those observed in monolayer cultures under basal conditions; however, no significant differences were observed in ON expression between epiphyseal and diaphyseal pellets (Fig. 3d), and neither OCN nor OSX were expressed (data not shown). Although expression of the chondrogenic gene COL2A1 was similar to that in monolayer cultures under basal conditions with a three-fold downregulation in diaphyseal pellets (Fig. 3e), no significant difference in SOX9 expression (Fig. 3e) was observed, and COL10A1 expression was reversed with a 2.5 fold downregulation in diaphyseal pellets (Fig. 3f).

**Fig. 4:**
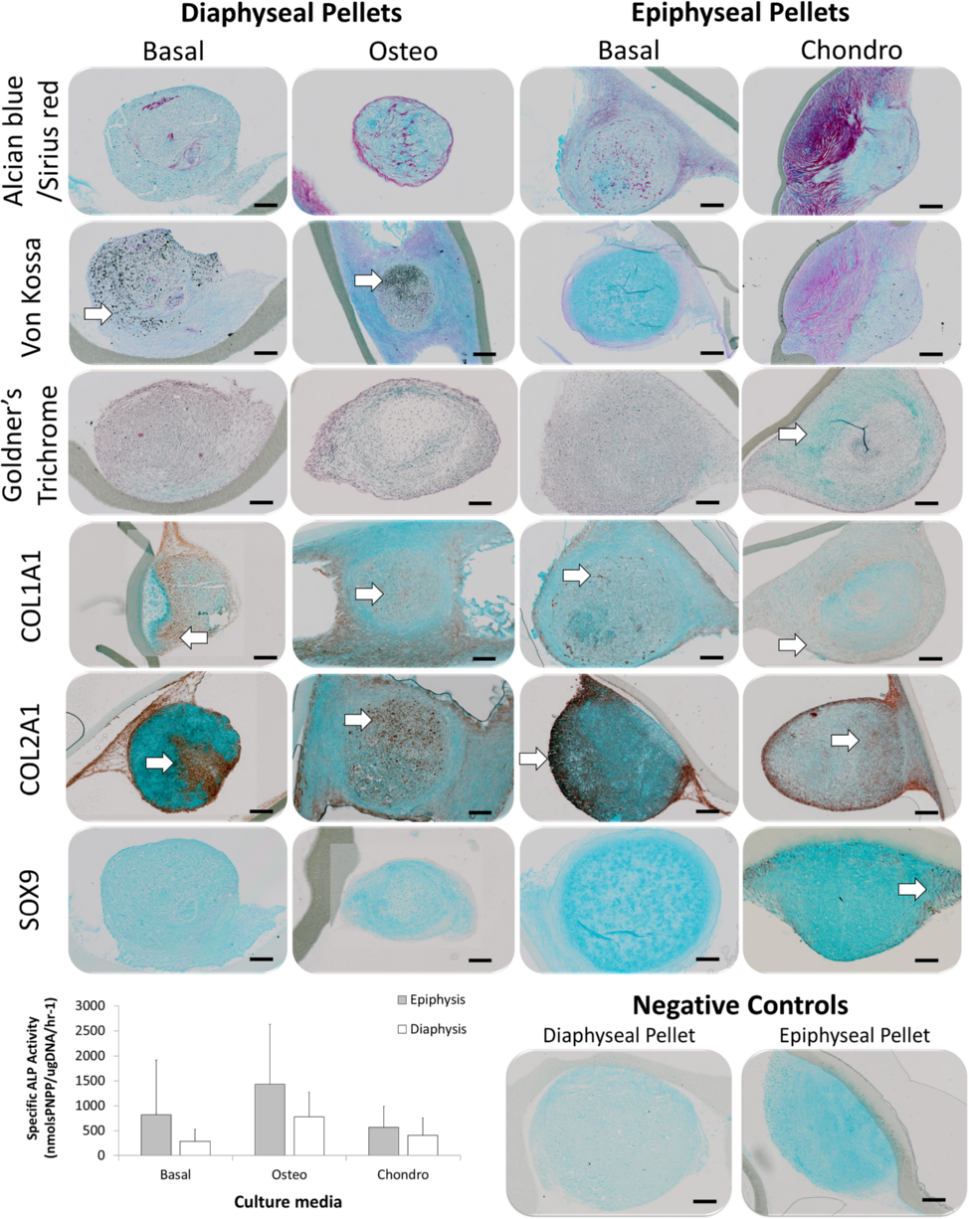
*In vitro* characterisation of epiphyseal and diaphyseal 3D pellet cultures. 2.5 × 10^5^ cells per pellet (P2) were cultured in basal medium for 48 h before transfer to differentiation medium for 21 days in organotypic culture. Diaphyseal pellets were cultured in basal and osteogenic medium, whilst epiphyseal pellets were cultured in basal and chondrogenic medium. Specific ALP activity was also assessed following 21 days culture. Scale bars measure 200 μm. (foetal samples, n = 6; 55, 55, 56, 63, 63 and 65 days post conception). *ALP* alkaline phosphatase

Histological analysis revealed greater collagen deposition following differentiation culture compared to basal conditions (Fig. 4). However, both basal and differentiation culture exhibited both COL1A1 and COL2A1 deposition in epiphyseal and diaphyseal pellets. Interestingly, only epiphyseal pellets cultured in chondrogenic media exhibited SOX9 expression and osteoid formation. In addition, only diaphyseal pellets, independent of culture medium, exhibited mineralised tissue formation.

#### *Ex vivo* differentiation capacity in epiphyseal and diaphyseal cell populations

**Fig. 5:**
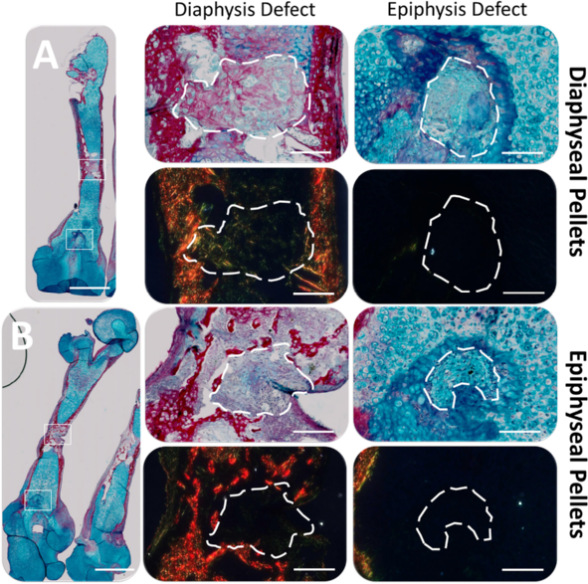
*Ex vivo* bone defect regeneration following implantation of diaphysis-derived **a** and epiphysis-derived **b** 3D cell pellets. Pellets (1.5 × 10^4^ cells per pellet [P2]) were implanted in drill defects (300 μm diameter) at both epiphyseal and diaphyseal locations on an embryonic day 11 chick femur, and incubated for 10 days in organotypic culture in basal medium. Femora were fixed, embedded in wax, sectioned and stained with Alcian blue/Sirius red and imaged for birefringence using polarising filters. Highly aligned collagen fibres in mineralised bone appeared red. Scale bars measure 1 mm (low magnification images) and 100 μm (high magnification images). (foetal samples, n = 4; 54, 59, 63 and 65 days post conception)

In marked contrast to control defects with no implants which remained empty (Additional file 3: Figure S2), all defects implanted with cell pellets exhibited new tissue formation (Fig. 5). Epiphyseal defects exhibited proteoglycan-rich tissue, whilst diaphyseal defects exhibited collagen deposition, independent of pellet type. Both epiphyseal (Fig. 5b) and diaphyseal pellets (Fig. 5a) demonstrated new matrix deposition relative to the implantation site, possibly due to local inductive signalling cues. Birefringence imaging showed that fibre alignment within new collagen deposits was comparable to that in native mineralised tissue (Fig. 5a).

### Characterisation of foetal femur cell populations isolated by Stro-1 immuno-selection

#### Human foetal femur development and Stro-1 expression

During development between 47 and 69 days post conception (age range of acquired samples in this study), human foetal femurs significantly increase in size with mineralised bone collar formation between 53 and 54 days (white arrows) and marrow invasion between 59 and 67 days (Additional file 5: Figure S4). White arrows point at the bone collar. Interestingly, Stro-1 expression in the femur loosely correlated with these developmental stages (Additional file 6: Figure S5). Early Stro-1 expression was observed around 47 days post conception prior to bone collar formation. Once bone collar formation began, Stro-1 expression was again observed between 55 and 61 days. Expression was not observed following marrow invasion.

#### 2D characterisation of Stro-1 immuno-selected cell populations

**Fig. 6:**
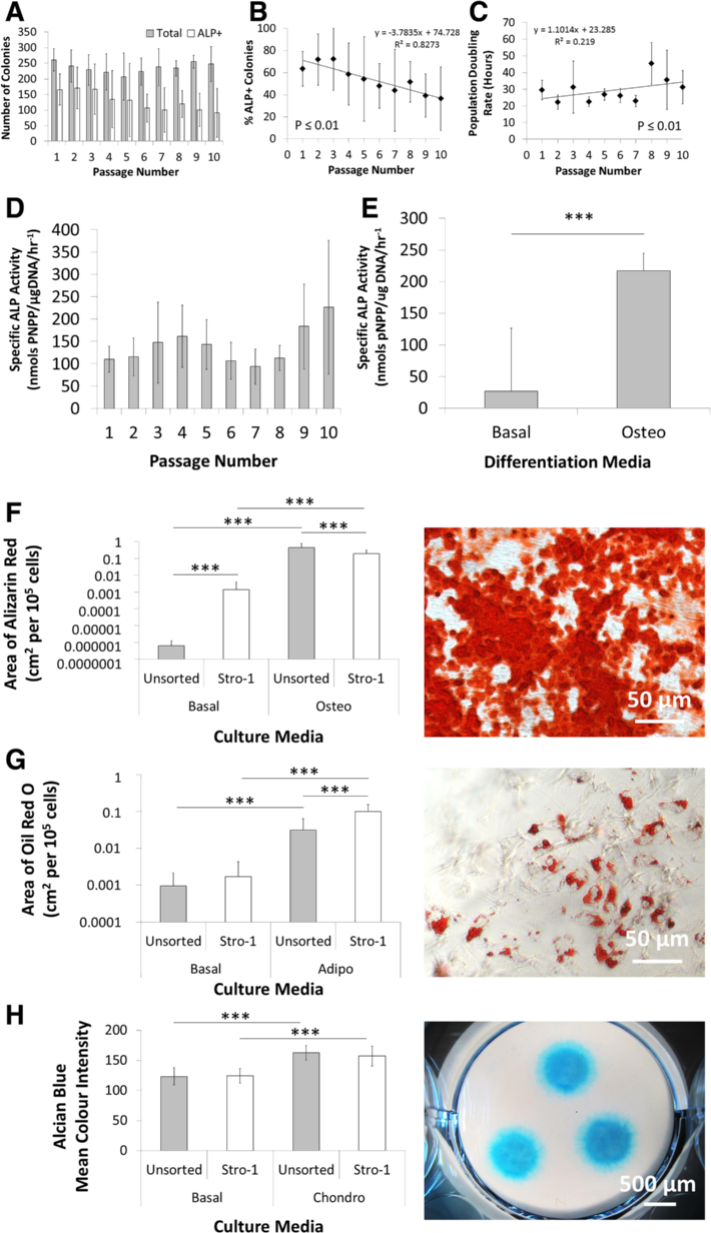
*In vitro* characterisation of Stro-1 immuno-selected populations. Isolated cell populations were seeded into CFU-F assay (1 × 10^2^ cells/cm^2^) over serial passage (P1 to P10) and cultured for 14 days in T25 cm^2^ flasks before fixation in 85 % ethanol and staining for ALP expression **a** (n = 4; 55, 55, 55 and 56 days of age foetal samples). ALP+ colonies are presented as a percentage of the total number of colonies counted **b**. Population doubling rate (**c**, foetal samples, n = 6; 55, 55, 55, 55, 56 and 56 days post conception), a measurement of proliferation, and specific ALP activity (**d**, foetal samples, n = 4; 55, 55, 56 and 56 days of age post conception) were also assessed over serial passage in monolayer cultures seeded at 1 × 10^3^ cells/cm^2^ (P2). Specific ALP activity was also assessed in cell populations (P2) following 14 days culture in basal or osteogenic medium (**e**, foetal samples, n = 3; 55, 56, and 59 days (Stro-1); 55, 55 and 61 days (unsorted) of age post conception). Additional monolayer cultures (1 × 10^3^ cells/cm^2^, P2) were treated with osteogenic and adipogenic (rosiglitazone) medium for 14 days, and stained with Alizarin red **f** and Oil Red O **g**, respectively. Micromass cultures (2.5 × 10^5^ cells per pellet, P2) were treated with chondrogenic medium for 14 days and stained with Alcian blue **h**. Control monolayers were cultured in basal medium. Representative images were of Stro-1 immuno-selected cultures in differentiation media. Error bars are SD. *** P ≤ 0.001 (foetal samples, n = 3; 55, 56 and 59 days (Stro-1); 55, 55 and 61 days (unsorted) of age post conception). *ALP* alkaline phosphatase

Stro-1 expression was observed in monolayer cultures over five serial passages, with a rapid reduction in Stro-1 expression after passage 6 and loss of expression after passage 7 (Additional file 7: Figure S6). No change in colony formation capacity over serial passage was observed (Fig. 6a). However, ALP+ colony formation capacity significantly (P ≤ 0.01) decreased over serial passage (Fig. 6b). Loss of ALP+ colony formation capacity appeared to correlate with significantly (P ≤ 0.01) increased population doubling rate; however, correlation was not specifically assessed (Fig. 6c). Interestingly, ALP activity appeared unchanged over serial passage (Fig. 6d), but significantly (P ≤ 0.001) increased following osteogenic culture (Fig. 6e). Significantly (P ≤ 0.001) enhanced calcium deposition and nodule formation was observed in both unsorted and Stro-1 immuno-selected populations following osteogenic compared to basal culture (Fig. 6f). Stro-1 immuno-selected populations exhibited significantly (P ≤ 0.001) increased nodule formation even in the absence of osteogenic induction. Both populations exhibited significantly increased (P ≤ 0.001) lipid formation following adipogenic culture, but Stro-1 immuno-selected populations exhibited significantly (P ≤ 0.001) higher lipid formation compared to unsorted populations (Fig. 6g). Proteoglycan deposition significantly (P ≤ 0.001) increased equally in both unsorted and Stro-1 immuno-selected populations following chondrogenic culture (Fig. 6h).

In comparison to adult Stro-1 immuno-selected populations which are known to exhibit decreased Stro-1 expression following *in vitro* expansion, foetal Stro-1 immuno-selected populations exhibited phenotype stability over several passages [60]. Furthermore, multi-lineage *in vitro* differentiation potential was shown to correlate with Stro-1 expression over early (P2) and late (P6) passage (Additional file 8: Figure S7). Osteogenic and chondrogenic differentiation were observed without induction, highlighting a preference towards these lineages. In basal cultures, adipogenic differentiation was observed at early (P2) but not late (P6) passage.

#### *Ex vivo* differentiation capacity in Stro-1 immuno-selected cell populations

**Fig. 7:**
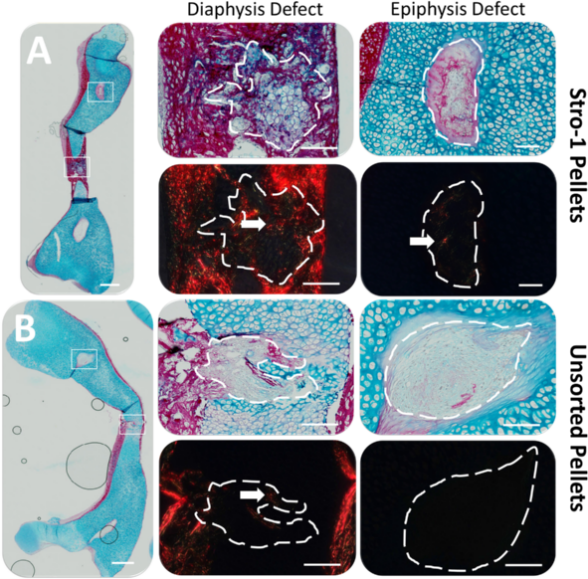
*Ex vivo* bone defect regeneration following implantation of Stro-1 **a** and unsorted **b** 3D pellets. Pellets (1.5 × 10^4^ cells per pellet [P2]) were implanted in drill defects (300 μm diameter) at both epiphyseal and diaphyseal locations on an embryonic day 11 chick femur, and incubated for 10 days in organotypic culture in basal medium. Femora were fixed, embedded in wax, sectioned and stained with Alcian blue/Sirius red and imaged for birefringence using polarising filters. Highly aligned collagen fibres in mineralised bone appeared red. Scale bars measure 1 mm (low magnification images) and 100 μm (high magnification images). (foetal samples, n = 3; 55, 55 and 56 days (Stro-1); 55, 55 and 61 days (unsorted) of age post conception)

Unsorted pellets exhibited negligible new matrix formation in both epiphyseal and diaphyseal defects (Fig. 7). In marked contrast, Stro-1 immuno-selected pellets exhibited collagen deposition in diaphyseal defects. Newly deposited collagen fibre alignment was similar to that of native mineralised tissues. New matrix formation in epiphyseal defects was reminiscent of diaphyseal tissue rather than epiphyseal tissue. Birefringence imaging revealed collagen alignment similar to that of native bone collar in Stro-1 immuno-selected pellets implanted in epiphyseal defects. Differentiation capacity and matrix deposition in unsorted and Stro-1 immuno-selected foetal populations were similar to that observed in adult populations (Additional file 9: Figure S8). Adult unsorted pellets exhibited minimum collagen deposition in both epiphyseal and diaphyseal defects, whilst adult Stro-1 pellets exhibited new collagen deposition with fibre alignment similar to that in native bone collar.

## Discussion

The current study has characterised both regional and immuno-selected skeletal cell populations derived from human foetal femora. These populations offer tools for both *in vitro* and *ex vivo* interrogation of the biological mechanisms controlling skeletal development and bone formation. Understanding these mechanisms using foetal populations as a developmental paradigm will inevitably inform successful *in vivo* tissue engineering strategies leading to improved bone formation and defect reparation downstream. Careful dissection through the metaphyses resulted in two regional populations, epiphyseal and diaphyseal cells. Distinction between the epiphysis and diaphysis is not clear as the metaphysis is a transitional zone between the two regions. The authors are aware that a simplified dissection approach to cell isolation may lead to cross-contamination of epiphyseal and diaphyseal populations. Indeed, further investigation revealed the presence of a shared sub-population of cells between both regions exhibiting Stro-1 expression, indicating a potential SSC source. Nevertheless, this study shows the osteochondral differentiation potential of these distinct foetal femur populations and their contribution to skeletal development and repair by means of a bone defect model.

2D *in vitro* characterisation revealed an osteogenic phenotype in diaphyseal populations (enhanced bone nodule formation) and a distinct chondrogenic phenotype in epiphyseal populations (enhanced proteoglycan and mucopolysaccharide deposition) indicative of the SSC maturation stages expected within these respective regions. Early markers of osteogenic differentiation capacity (ALP, COL1A1, RUNX2 and OSX) were observed to be higher in diaphyseal populations. As would be expected with early osteogenic differentiation, expression of late markers such as OCN, was not increased. However, an alternative mid-late osteogenic marker ON *was* significantly increased indicating the presence of a sub-population of diaphyseal cells that had become fully differentiated and functional osteoblasts in the 14 day culture period. It is important to note here that although OCN expression was not significantly increased in diaphyseal populations, expression was still observed confirming the presence of mature osteoblasts. Expression profiles of early (increased SOX9 and COL2A1) and late (reduced COL10A1) chondrogenic genes in epiphyseal populations indicated elevated early chondrocyte differentiation. Higher COL10A1 expression in diaphyseal populations indicated the presence of hypertrophic chondrocytes, important in the formation of mineralised cartilage. Similar trends were observed following 3D pellet culture for osteogenic genes including ALP, COL1A1 and RUNX2. However, unlike monolayer cultures, expression of mid to late osteogenic genes was not observed indicating the absence of terminal differentiation. Indeed, expression of chondrogenic gene SOX9 was not significantly different between epiphyseal and diaphyseal populations suggesting cartilaginous and pre-osteoid formation was dominant. In support, COL10A1 expression was reduced in diaphyseal populations highlighting the lack of hypertrophic chondrocytes important for mineralisation. Critically, the similarities and differences observed may be due to separate maturation stages within the respective populations. As such, further investigation over serial passage would be required to assess the stability of epiphyseal and diaphyseal population phenotypes.

Histological analysis revealed mineralisation in diaphyseal pellets and SOX9 expression in epiphyseal pellets. Counter-intuitively, COL1A1 and COL2A1 deposition was observed in both epiphyseal and diaphyseal pellets, although a shared sub-population and rapid developmental background offers some explanation as to why both regional derived populations exhibited expression of both osteogenic and chondrogenic markers. It is important to note that cross-contamination of populations during isolation may also explain the dual expression of COL1A1 and COL2A1. Both cell origin and implantation site correlated with defect regeneration capacity; epiphyseal and diaphyseal populations exhibited differentiation plasticity dependent upon their environment, suggesting the presence of a shared inducible sub-population of progenitor cells.

Developmental stage-dependent Stro-1 expression (adult SSC marker [5, 65, 66]) was observed in human foetal femora in the epiphyses and the diaphysis, supporting the presence of a shared SSC sub-population. Interestingly, Stro-1 immuno-selected populations exhibited phenotype stability over prolonged passage, unlike their adult Stro-1 counterparts which lose expression after a single *in vitro* passage. Encouragingly, Stro-1 expression correlated with multi-lineage differentiation potential at early (P2) and late (P6) passage. Expression also appeared to correlate with decreased cell proliferation and ALP+ colony formation, indicating possible emergence of a clonogenic fibroblastic phenotype, often observed in prolonged *in vitro* cultures [67].

Without exogenous induction, Stro-1 immuno-selected populations exhibited strong differentiation potential towards the osteogenic lineage; this may explain why Stro-1 implants displayed only bone-like tissue formation. Unsorted populations demonstrated similar differentiation potential to Stro-1 immuno-selected populations. However, this did not translate to functional tissue regeneration, evidenced by reduced new collagen and proteoglycan deposition. This may be due to the lack of inductive signals *in vivo* for unsorted populations, whereas Stro-1 immuno-selected populations represent cell populations already committed to the osteochondral differentiation path. Foetal Stro-1 pellets demonstrated similar differentiation capacity to their adult Stro-1 counterparts; both exhibited osteogenic differentiation potential independent of implantation site. This may be due to underlying mechanisms and environmental factors in 3D pellet cultures that drive differentiation along the osteogenic lineage.

Interestingly, both regional populations and Stro-1 immuno-selected populations isolated from human foetal femora clearly demonstrated capacity for multi-lineage differentiation and formation of new matrix reminiscent of bone, cartilage and fat. However, to fully determine their capacity for functional tissue regeneration and, therefore, their application as an investigative tool, further *in vivo* experimentation is required.

## Conclusions

The current study demonstrates isolation of distinct cell populations from human foetal femora according to anatomical location and immuno-selection. Regionally derived populations from the epiphyses and diaphysis offer potential cell sources for routine chondrogenic and osteogenic differentiation, respectively. A shared SSC sub-population, however, affords both epiphyseal and diaphyseal populations a degree of differentiation plasticity. Stro-1 enrichment isolated an SSC alternative to their conventional adult counterparts. However, to fully understand the tissue regenerative capacity of these distinct populations, further investigation is required to assess functional tissue formation *in vivo*. The work presented here shows that foetal femur-derived populations offer investigative tools with which to understand biological mechanisms involved in skeletal development and their translation to regenerative medicine. Furthermore, *ex vivo* assessment of these populations provides a novel system with which to screen potential tissue engineering strategies prior to time consuming (and expensive) *in vivo* studies.

## Additional files

Additional file 1: Figure S1. Human foetal femur following dissection and removal of surrounding soft tissues (**A**) and an x-ray radiograph (**B**). Red lines depict metaphyseal regions. White lines depict the epiphyseal and diaphyseal regions. (foetal sample; 65 days post conception). (JPEG 831 kb) Additional file 2: Table S1. Forward and reverse primer sequences used for quantitative RT-PCR analysis. (JPEG 1068 kb) Additional file 3: Figure S2. Control defect generation on embryonic day 11 chick femora in organotypic culture. Drill defects (300 μm diameter) were created along the length of the femur and restricted to the epiphyses and diaphysis prior to pellet implantation. Control sham defects were created and chick femora were organotypically cultured for 10 days. (JPEG 1137 kb) Additional file 4: Figure S3. Correlation between seeded cell number and 3D pellet diameter. Human foetal femur derived cells were suspended in basal medium and centrifuged at 1,000 rpm for 4 min to form pellets. 3D pellets were subsequently cultured in basal medium for 48 h. Pellets were formed from a range of cell numbers (1 × 10^4^ to 1 × 10^6^ cells). Following pellet formation and incubation, pellets were imaged by light microscopy and their diameter recorded. Error bars show SD. (foetal samples, n = 3; 54, 54 and 63 days of age post conception). (JPEG 381 kb) Additional file 5: Figure S4. Development of human foetal femora and bone collar formation. Human foetal femora covering a range of developmental stages from 47 to 69 days post conception were fixed, embedded in wax, sectioned and stained with Alcian blue/Sirius red. Femora increase significantly in size and exhibit both mineralisation and marrow cavity formation. Scale bars measure 100 μm (high magnification images) and 500 μm (low magnification images). (JPEG 3785 kb) Additional file 6: Figure S5. Development of human foetal femora and Stro-1 expression. Human foetal femora covering a range of developmental stages from 47 to 69 days post conception were fixed, embedded in wax, sectioned and Stro-1 immuno-labelled. Stro-1 appeared dependent on developmental stage with expression around 47 days and 59 to 61 days post conception, largely restricted to the diaphysis (depicted as bright brown stain). High magnification images of the femur at 47 days clearly show Stro-1 expression in the diaphysis but not in the epiphysis. Negative controls were stained without the primary antibody. Scale bars in epiphysis and diaphysis individual images measure 100 μm. Scale bars in the femur overview images measure 500 μm. (JPEG 3030 kb) Additional file 7: Figure S6. Maintenance of Stro-1 expression following MACS isolation over serial passage *in vitro*. Isolated cells were seeded into 12-well plates (1 × 10^3^ cells/cm^2^) at each consecutive passage (P1 to P10), and cultured for three to five days (for cell adhesion and proliferation) before fixation in 4 % PFA. Monolayers labelled for Stro-1 expression, counterstained with DAPI were imaged by fluorescence microscopy. *Left windows* show fluorescence images and *right windows* show the same images in brightfield. Scale bars measure 50 μm. (foetal samples, n = 3; 55, 55 and 56 days post conception). (JPEG 1864 kb) Additional file 8: Figure S7. Differentiation potential of Stro-1 immuno-selected populations from human foetal femora over extended passage. Isolated cells were seeded (1 × 10^3^ cells/cm^2^) as monolayer cultures and expanded to P2 (*top two rows*) and P6 (*bottom two rows*) before differentiation in osteogenic and adipogenic (indomethacin) medium for 14 and 28 days, respectively. Micromass pellets (2.5 × 10^5^ cells per pellet) were formed in basal medium for 1 h before culture in chondrogenic medium for 21 days. Control cultures were treated with basal medium. Osteogenesis was assessed by ALP expression, chondrogenesis by proteoglycan deposition (Alcian blue staining), and adipogenesis by lipid deposition (Oil Red O staining). (foetal samples, n = 3; 55, 55 and 55 days post conception). (JPEG 3635 kb) Additional file 9: Figure S8.
*Ex vivo* bone defect regeneration following implantation of Stro-1 immuno-selected and unsorted cell pellets derived from adult human bone marrow. Pellets (1.5 × 10^4^ cells per pellet (P2)) were implanted in drill defects (300 μm diameter) at both epiphyseal and diaphyseal locations on an embryonic day 11 chick femur, and incubated for 10 days in organotypic culture in basal medium. Femora were fixed, embedded in wax, sectioned and stained with Alcian blue/Sirius red and imaged for birefringence using polarising filters. Highly aligned collagen fibres in mineralised bone red. Scale bars measure 100 μm. (n = 5; F54, F67, F71, M60 and M78 (Stro-1); F52, M46, M67, M75 and M78 (unsorted)) M = male; F = female. (JPEG 1702 kb)

## Abbreviations

2D: Two dimensional
3D: Three dimensional
A/S: Alcian blue/Sirius red
ALP: Alkaline phosphatase
Asc: Ascorbate-2-phosphate
BSA: Bovine serum albumin
CFU-F: Colony forming unit-fibroblastic
DAPI: 4′,6-diamidino-2-phenylindole
Dex: Dexamethasone
EDTA: Ethylenediaminetetraacetic acid
FCS: Foetal calf serum
HA-PLA: Hydroxyapatite-loaded poly - lactic acid
HBMSC: Human bone marrow stromal cell
IBMX: Isobutyl-1-methylxanthine
ITS: Insulin/transferrin/sodium selenite
MACS: Magnetic activated cell sorting
MSC: Mesenchymal stem cell
OCN: Osteocalcin
ON: Osteonectin
OSX: Osterix
P1/P2/P6: Passage 1/2/6
PBS: Phosphate buffered saline
PCR: Polymerase chain reaction
PFA: Paraformaldehyde
PTFE: Polytetrafluoroethylene
RT-qPCR: Reverse transcription-quantitative PCR
SD: Standard deviation
SSC: Skeletal stem cell
UV: ultraviolet
α-MEM: Modified Eagle’s medium - alpha
