# Supplementary figures and images for: Regionally-derived cell populations and skeletal stem cells from human foetal femora exhibit specific osteochondral and multi-lineage differentiation capacity in vitro and ex vivo
Source: Stem Cell Res Ther. 2015 Dec 18;6:251. doi: 10.1186/s13287-015-0247-2 (PMC4683700; doi:10.1186/s13287-015-0247-2)

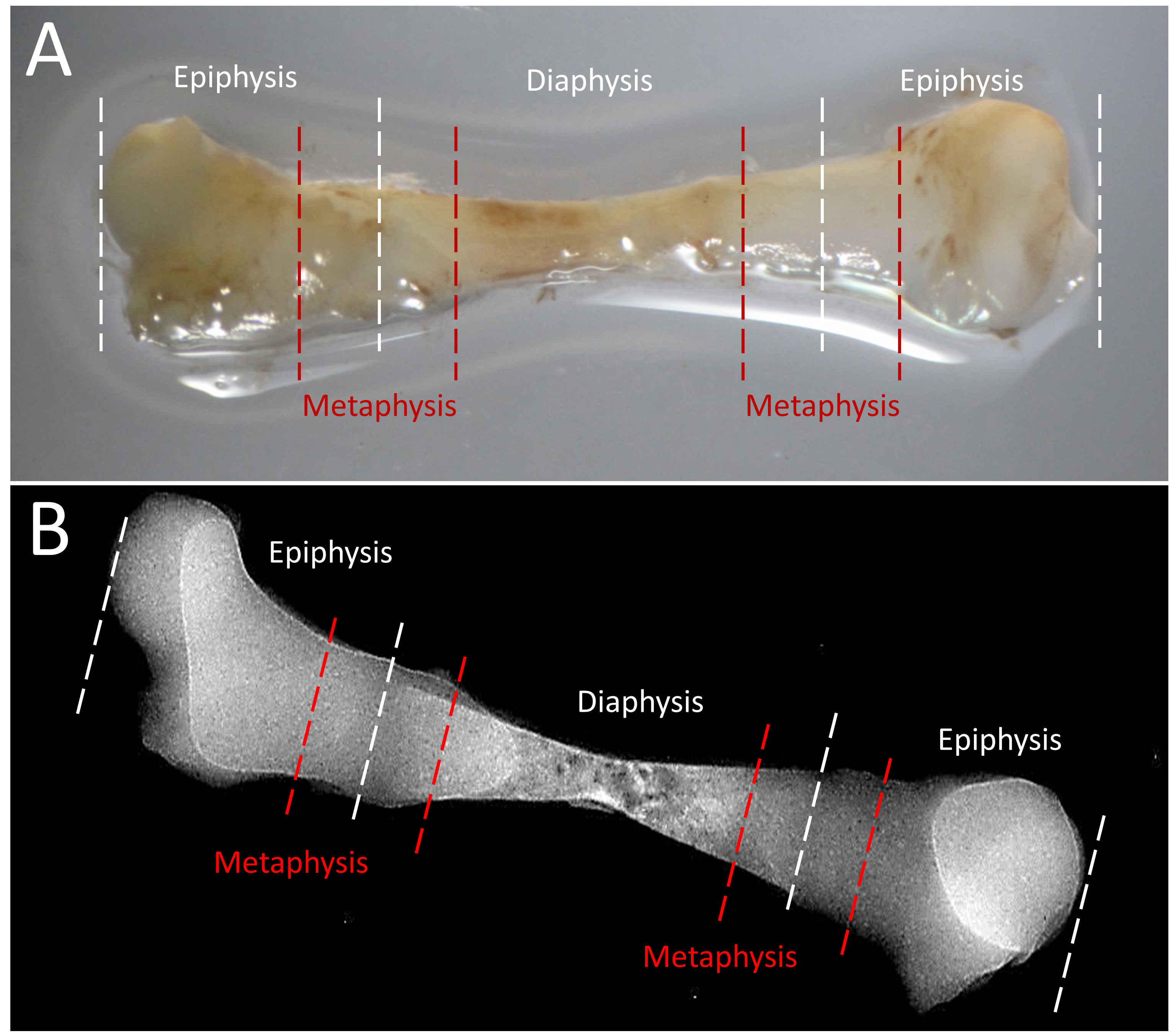

Supplement: Additional file 1: Figure S1. — Human foetal femur following dissection and removal of surrounding soft tissues (A) and an x-ray radiograph (B). Red lines depict metaphyseal regions. White lines depict the epiphyseal and diaphyseal regions. (foetal sample; 65 days post conception). (JPEG 831 kb) [file 13287_2015_247_MOESM1_ESM.jpeg]

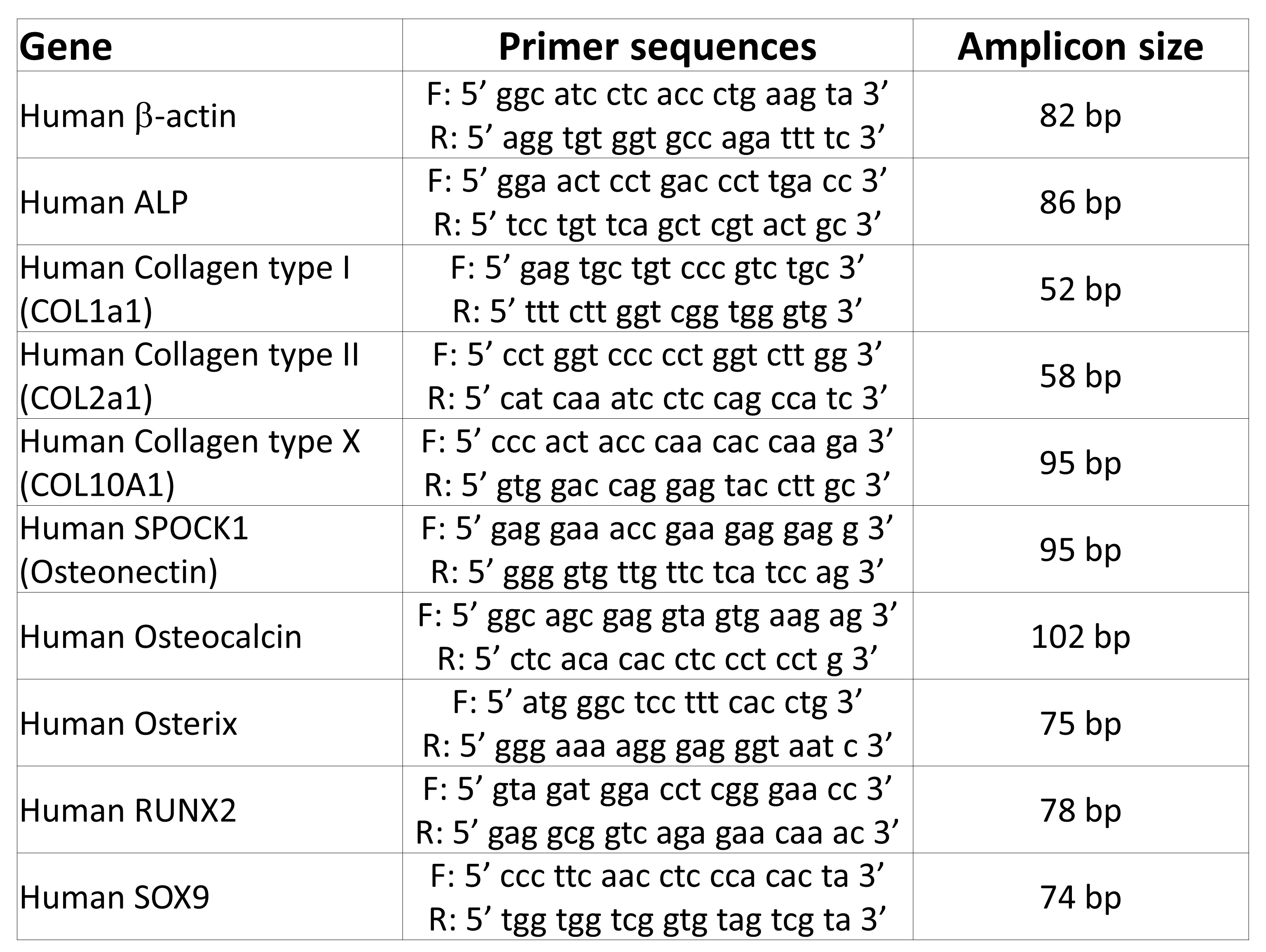

Supplement: Additional file 2: Table S1. — Forward and reverse primer sequences used for quantitative RT-PCR analysis. (JPEG 1068 kb) [file 13287_2015_247_MOESM2_ESM.jpeg]

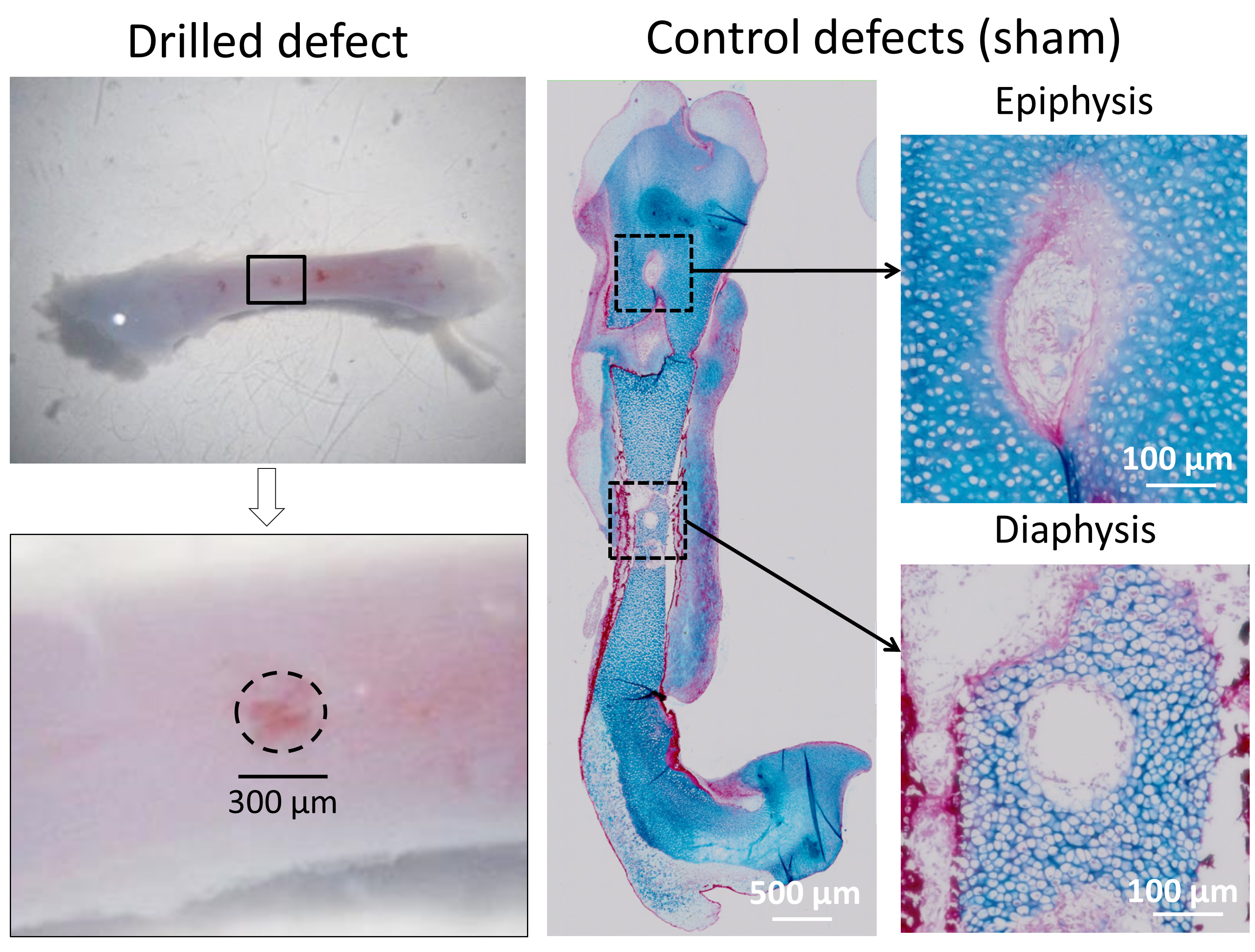

Supplement: Additional file 3: Figure S2. — Control defect generation on embryonic day 11 chick femora in organotypic culture. Drill defects (300 μm diameter) were created along the length of the femur and restricted to the epiphyses and diaphysis prior to pellet implantation. Control sham defects were created and chick femora were organotypically cultured for 10 days. (JPEG 1137 kb) [file 13287_2015_247_MOESM3_ESM.jpeg]

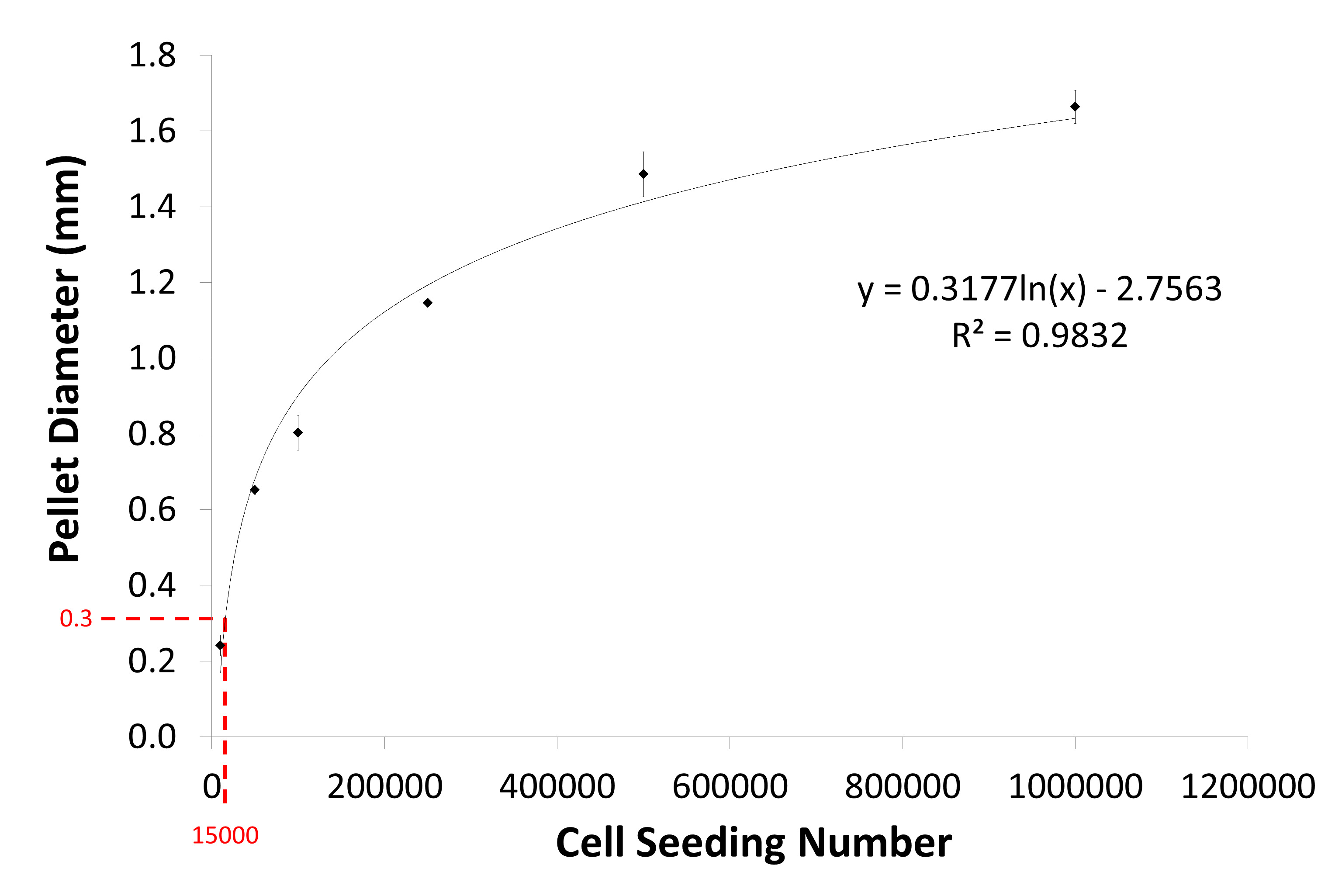

Supplement: Additional file 4: Figure S3. — Correlation between seeded cell number and 3D pellet diameter. Human foetal femur derived cells were suspended in basal medium and centrifuged at 1,000 rpm for 4 min to form pellets. 3D pellets were subsequently cultured in basal medium for 48 h. Pellets were formed from a range of cell numbers (1 × 10^4^ to 1 × 10^6^ cells). Following pellet formation and incubation, pellets were imaged by light microscopy and their diameter recorded. Error bars show SD. (foetal samples, n = 3; 54, 54 and 63 days of age post conception). (JPEG 381 kb) [file 13287_2015_247_MOESM4_ESM.jpeg]

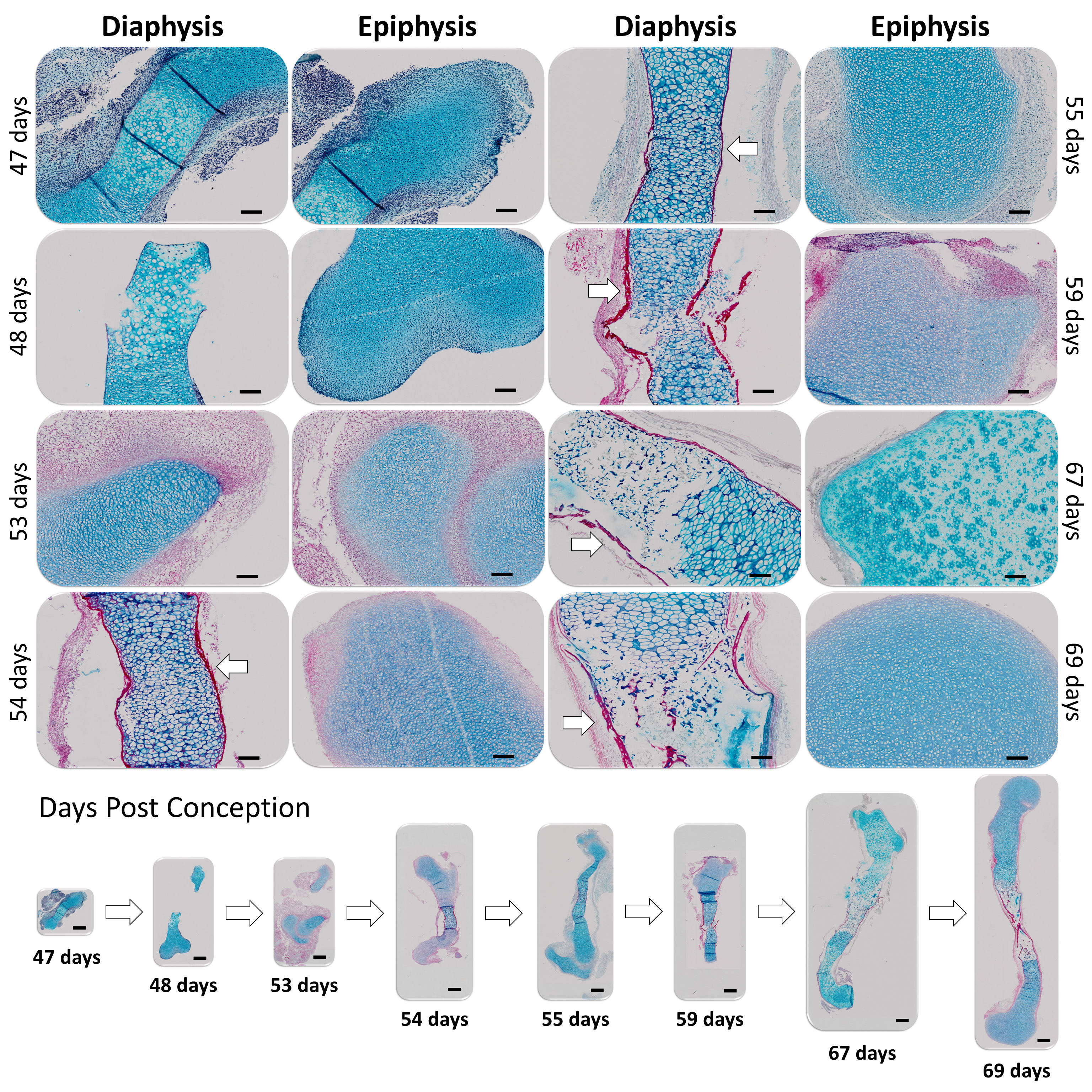

Supplement: Additional file 5: Figure S4. — Development of human foetal femora and bone collar formation. Human foetal femora covering a range of developmental stages from 47 to 69 days post conception were fixed, embedded in wax, sectioned and stained with Alcian blue/Sirius red. Femora increase significantly in size and exhibit both mineralisation and marrow cavity formation. Scale bars measure 100 μm (high magnification images) and 500 μm (low magnification images). (JPEG 3785 kb) [file 13287_2015_247_MOESM5_ESM.jpeg]

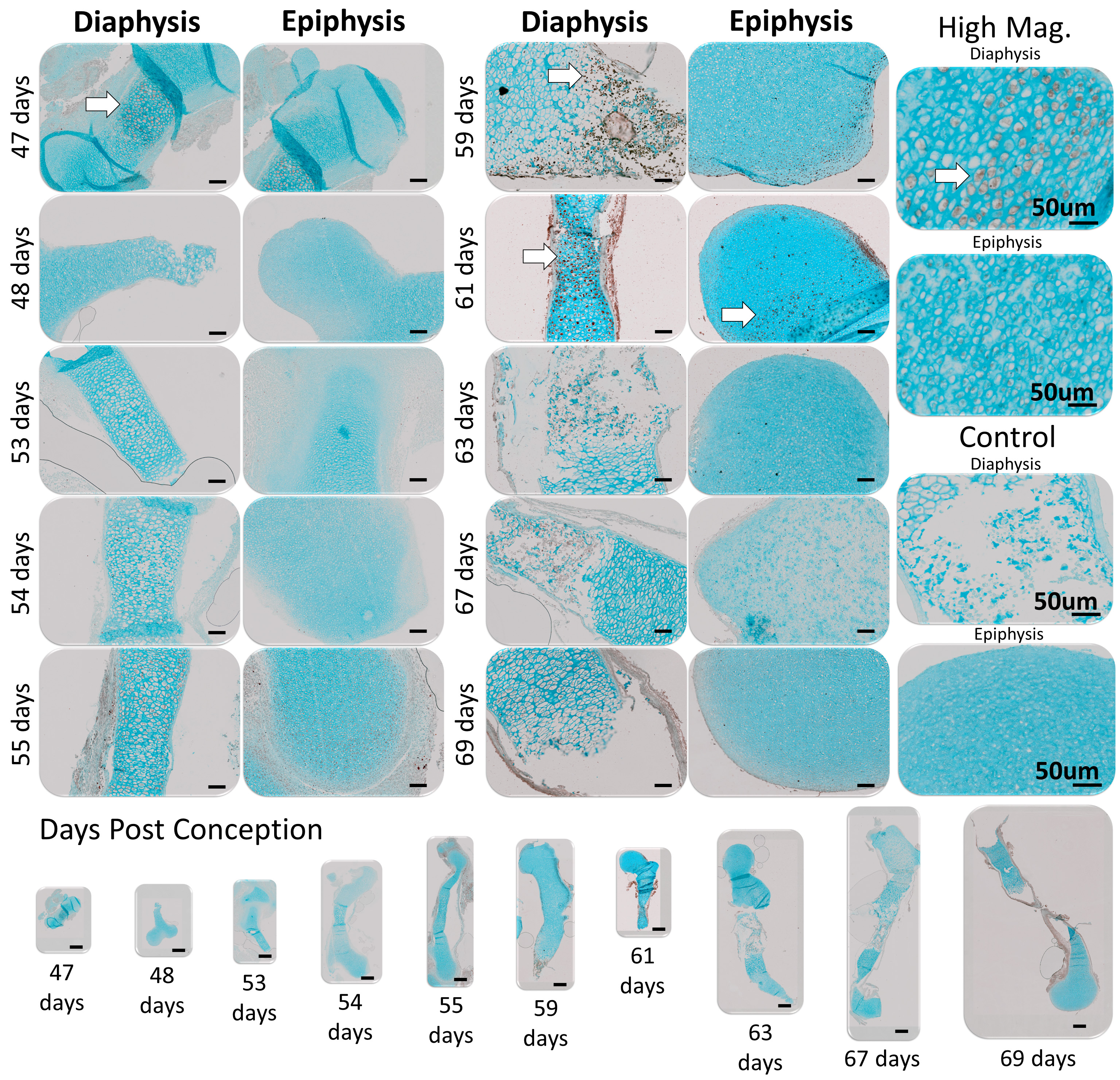

Supplement: Additional file 6: Figure S5. — Development of human foetal femora and Stro-1 expression. Human foetal femora covering a range of developmental stages from 47 to 69 days post conception were fixed, embedded in wax, sectioned and Stro-1 immuno-labelled. Stro-1 appeared dependent on developmental stage with expression around 47 days and 59 to 61 days post conception, largely restricted to the diaphysis (depicted as bright brown stain). High magnification images of the femur at 47 days clearly show Stro-1 expression in the diaphysis but not in the epiphysis. Negative controls were stained without the primary antibody. Scale bars in epiphysis and diaphysis individual images measure 100 μm. Scale bars in the femur overview images measure 500 μm. (JPEG 3030 kb) [file 13287_2015_247_MOESM6_ESM.jpeg]

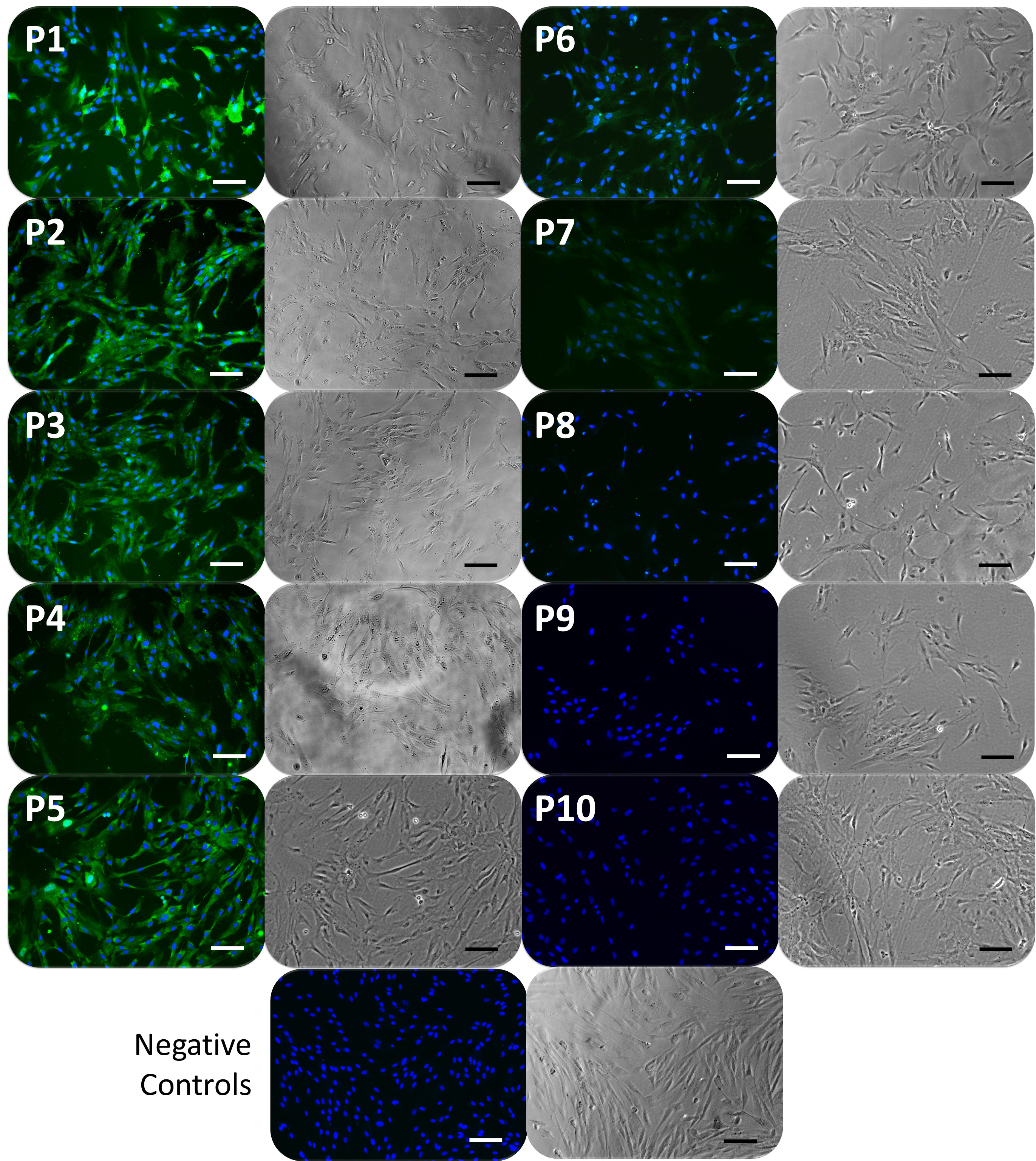

Supplement: Additional file 7: Figure S6. — Maintenance of Stro-1 expression following MACS isolation over serial passage in vitro. Isolated cells were seeded into 12-well plates (1 × 10^3^ cells/cm2) at each consecutive passage (P1 to P10), and cultured for three to five days (for cell adhesion and proliferation) before fixation in 4 % PFA. Monolayers labelled for Stro-1 expression, counterstained with DAPI were imaged by fluorescence microscopy. Left windows show fluorescence images and right windows show the same images in brightfield. Scale bars measure 50 μm. (foetal samples, n = 3; 55, 55 and 56 days post conception). (JPEG 1864 kb) [file 13287_2015_247_MOESM7_ESM.jpeg]

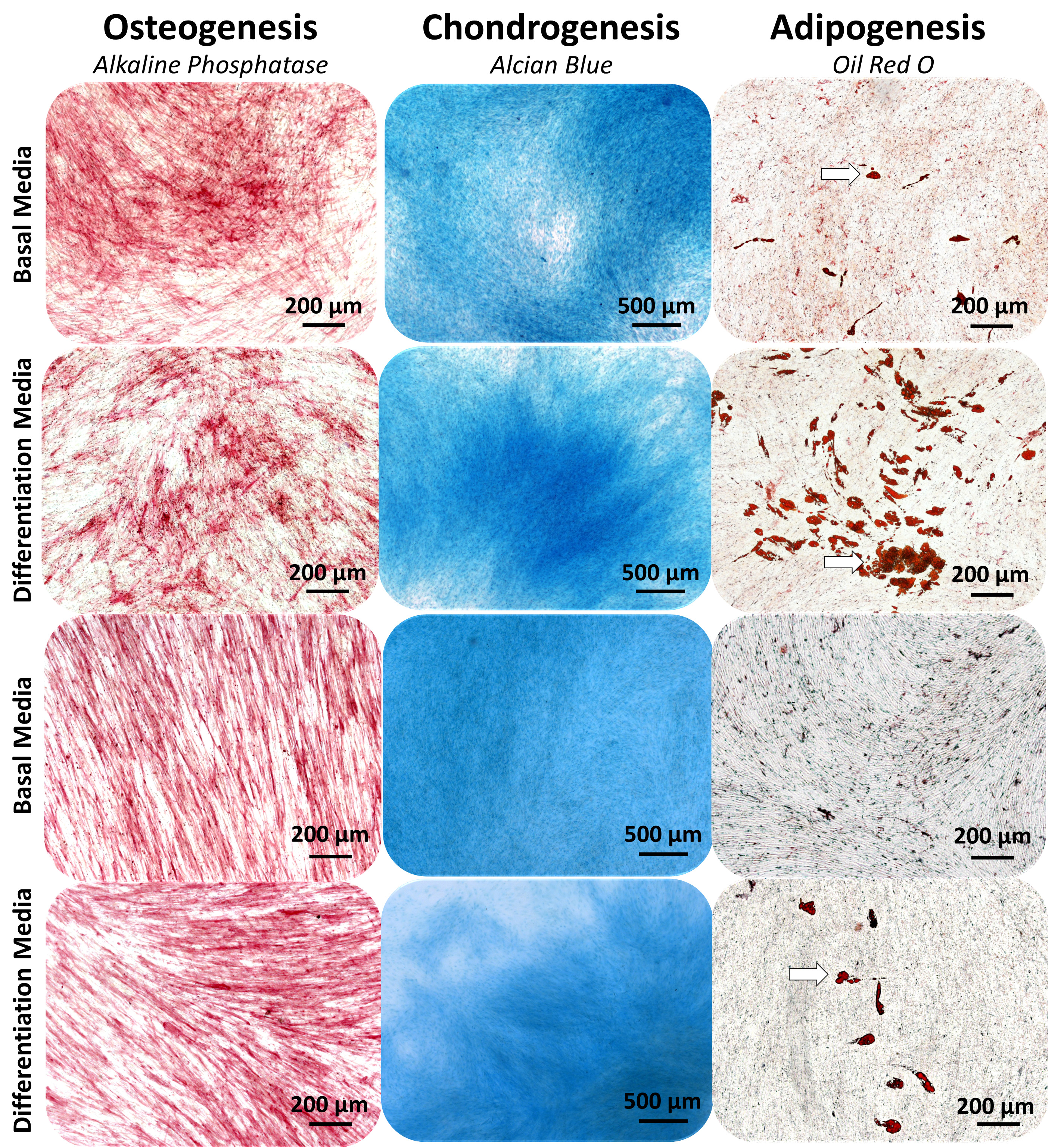

Supplement: Additional file 8: Figure S7. — Differentiation potential of Stro-1 immuno-selected populations from human foetal femora over extended passage. Isolated cells were seeded (1 × 10^3^ cells/cm2) as monolayer cultures and expanded to P2 (top two rows) and P6 (bottom two rows) before differentiation in osteogenic and adipogenic (indomethacin) medium for 14 and 28 days, respectively. Micromass pellets (2.5 × 10^5^ cells per pellet) were formed in basal medium for 1 h before culture in chondrogenic medium for 21 days. Control cultures were treated with basal medium. Osteogenesis was assessed by ALP expression, chondrogenesis by proteoglycan deposition (Alcian blue staining), and adipogenesis by lipid deposition (Oil Red O staining). (foetal samples, n = 3; 55, 55 and 55 days post conception). (JPEG 3635 kb) [file 13287_2015_247_MOESM8_ESM.jpeg]

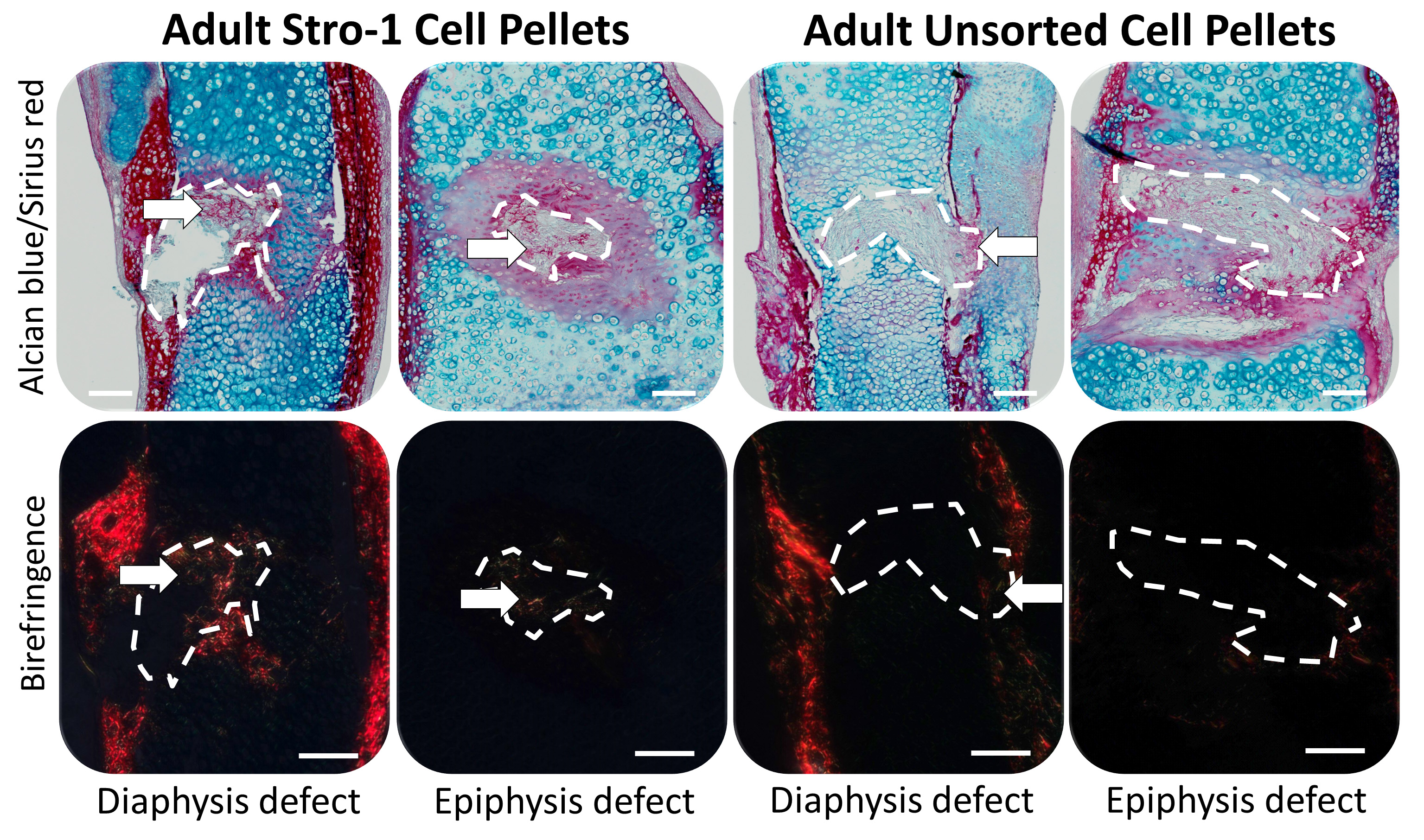

Supplement: Additional file 9: Figure S8. — Ex vivo bone defect regeneration following implantation of Stro-1 immuno-selected and unsorted cell pellets derived from adult human bone marrow. Pellets (1.5 × 10^4^ cells per pellet (P2)) were implanted in drill defects (300 μm diameter) at both epiphyseal and diaphyseal locations on an embryonic day 11 chick femur, and incubated for 10 days in organotypic culture in basal medium. Femora were fixed, embedded in wax, sectioned and stained with Alcian blue/Sirius red and imaged for birefringence using polarising filters. Highly aligned collagen fibres in mineralised bone red. Scale bars measure 100 μm. (n = 5; F54, F67, F71, M60 and M78 (Stro-1); F52, M46, M67, M75 and M78 (unsorted)) M = male; F = female. (JPEG 1702 kb) [file 13287_2015_247_MOESM9_ESM.jpeg]
